# Rhythm disturbance in osteoarthritis

**DOI:** 10.1186/s12964-022-00891-7

**Published:** 2022-05-24

**Authors:** Ze Du, Xuanhe You, Diwei Wu, Shishu Huang, Zongke Zhou

**Affiliations:** 1 Department of Orthopedics, West China Hospital, Sichuan University, 610041 Chengdu, China; 2 Department of Orthopedics and Research institute of Orthopedics, West China Hospital, Sichuan University, Chengdu, 610041 China

**Keywords:** Osteoarthritis, Rhythm, Cartilage, Subchondral bone, Synovium, Skeletal muscle

## Abstract

Osteoarthritis (OA) is one of the main causes of disabilities among older people. To date, multiple disease-related molecular networks in OA have been identified, including abnormal mechanical loadings and local inflammation. These pathways have not, however, properly elucidated the mechanism of OA progression. Recently, sufficient evidence has suggested that rhythmic disturbances in the central nervous system (CNS) and local joint tissues affect the homeostasis of joint and can escalate pathological changes of OA. This is accompanied with an exacerbation of joint symptoms that interfere with the rhythm of CNS in reverse. Eventually, these processes aggravate OA progression. At present, the crosstalk between joint tissues and biological rhythm remains poorly understood. As such, the mechanisms of rhythm changes in joint tissues are worth study; in particular, research on the effect of rhythmic genes on metabolism and inflammation would facilitate the understanding of the natural rhythms of joint tissues and the OA pathology resulting from rhythm disturbance.

**Video Abstract**

**Supplementary Information:**

The online version contains supplementary material available at 10.1186/s12964-022-00891-7.

## Background

With the increasing number of obese and older people, osteoarthritis (OA) has become one of the severe causes of disability among the elderly. OA affects nearly 250 million people worldwide, with corresponding medical costs having risen to 1% to 2.5% of gross domestic product in high-income countries [1]. OA is regarded as a disease associated to aging and correlated with gender, obesity, joint trauma and peripheral muscle weakness [2, 3]. The pathologic changes of OA slowly progress and are irreversible, and they are characterized by a deficiency of vasculature in cartilage, a low proliferation of chondrocytes and a reduction of matrix genesis [4]. Although clinical anti-inflammation and cartilage matrix-forming approaches have led to the relief of OA pain, these approaches have neither repaired cartilage from damage nor prevented OA progression. This indicates that the key mechanism of OA etiology is still unclear [5].

It has been reported that a 24-h diurnal rhythm is crucial in most physiological processes within the human brain and body [6]. A normal biological rhythm is essential for maintaining the homeostasis of peripheral tissues, such as skeletal muscle, bone, synovium and cartilage, and it has the following characteristics. First, these rhythms in peripheral tissues occur at their own pace through the oscillatory expressions of intrinsic rhythmic genes, such as brain and muscle arnt-like protein 1 (*Bmal1)*, circadian locomotor output cycles kaput (*Clock*), period 1 (*Per1*), period 2 (*Per2*) and cryptochrome 1 (*Cry1*). Second, rhythmic genes in peripheral tissues are adjusted by the central rhythmic oscillation system and, in particular, the suprachiasmatic nucleus (SCN) [7]. In addition, the rhythms of peripheral tissues are affected by outer and inner environmental changes, including the alternations of humidity and temperature by season and by day [8]. Interestingly, such biological rhythms exist in the musculoskeletal system. For instance, the roles of anabolism and catabolism have demonstrated rhythmic oscillations in cartilage. A group of factors in serum and urine have been identified in cartilage metabolic rhythms, including serum cartilage oligomeric matrix protein (COMP), hyaluronan (HA), keratan sulfate (KS5D4), transforming growth factor b1 (TGFB1), urinal C-telopeptide of Collagen II (CTXII), serum procollagen type IIA N-terminal propeptide (PIIANP) and type II collagen helical peptide (HELIXII) [9–11]. These cartilage metabolic factors peak in the morning, indicating a high level of cartilage metabolism during the night. Additionally, multiple rhythmic genes have been identified in the musculoskeletal system as maintaining normal biological rhythms, including *Bmal1*, *Per1*, *Per2*, *Cry1*, nuclear receptor subfamily 1 group D member 1 (*Nr1d1*) and nuclear receptor subfamily 1 group D member 2 (*Nr1d2*) [12]. These genes work together to maintain the homeostasis of cartilage by controlling the metabolic and inflammatory pathways in chondrocytes.

OA is an example of a condition causing chronic primary musculoskeletal pain and is thought to be associated with daily rhythm. The incidence and symptoms of OA are closely associated with rhythms. The risk of OA is tightly related to rhythm disturbance. “Shift work” refers to work schedules that deviate from standard working hours and include evening shifts, rotating shifts and night shifts. The incidence of OA usually increases with prolonged periods of shift work. On the contrary, the risk of OA decreases with shortened periods of shift work [13]. On the other hand, the common complaints of OA, stiffness and pain, are tightly rhythm related [14]. In general, OA joint pain has been confirmed to be more severe in the late afternoon than in the morning due to the activity of the day. Disregarding activity, however, OA joint pain seems to be more severe in the morning and causes worse posture balance in the late morning than in the afternoon among OA patients [15]. On the other hand, these symptoms of OA are affected by aberrant sleep/activity rhythms. When daily rhythm is disturbed by poor sleeping, OA patients develop heightened pain and fatigue conditions during the following day [16].

As such, we will discuss the relationship between the rhythmic disturbance and OA, including the abnormal expression of rhythmic genes, oscillatory secreted hormones and other possibly rhythmic factors. Additionally, the network of rhythmic disorders in joint tissues is identified, as it may provide new targets for the treatment of OA symptoms and the inhibition of OA progression. Altogether, we hope this discussion will shed new light on the interaction between biological rhythm disturbances and OA development.

## Normal joint rhythms

Cartilage, synovium, bone and skeletal muscle are reported to have regular rhythms for maintaining joint homeostasis (Table 1). And knockout of rhythmic genes can change normal cell phenotype in periarticular tissues (Table 2).

**Table 1:** Function and targeted genes of intrinsic rhythmic genes in joint

	Rhythmic genes	Target		Function	Reference
		Activate	Depress		
Cartilage	*Bmal1*	*Tgfb*, *Clock*, *Sox9*	*Mmp13*, *Adamts5*, *Nfkb*	Chondrocyte hypertrophy defending; Cartilage degeneration inhibition	[23, 49]
	*Clock*	*Per2*, *Dbp*, *E4bp4*, *Adamts4*	*Mmp14*, *Il6*, *Il1b*, *Mcp1*	Cartilage rhythm maintaining; Anti-inflammation in cartilage	[19]
	*Cry2*		*Nr1d1*, *Nr1d2*, *Dbp*, *Tef*	Cartilage rhythm maintaining	[24]
	*Per2*	*Tgfb*, *Mmp13*, *Adamts5*	*Bmal1*, *Sox9*	Cartilage degeneration activation; Cartilage generation inhibition	[49]
Synovium	*Bmal1*	*Clock*, *Nr1d1*, *Il10*, *Ifng*, *Il13*	*Il6*, *Cxcl1*, *Ccl2*, *Cxcl5*	Synovium rhythm maintaining; Anti-inflammation in synovium	[32]
Subchondral bone	*Bmal1*	Nfatc1*	Mmp9*, CatK*, Trap*, Rank*, Calcr*, Rankl*	Bone mineral density; Bone volume maintaining; Osteoclast volume maintaining	[29, 31]
	*Clock* *	*Bmal1*, Fabp4*		Subchondral bone rhythm maintaining; Adipogenesis of bone marrow MSC	[28]
	Per2*	Per1*, *C/ebpalpha* *		Subchondral bone rhythm maintaining; Adipogenesis of bone marrow MSC	
	Gsk3b*	Fabp4*, Pparg*, *C/ebpalpha* *, Alp*		Adipogenesis of bone marrow MSC; Osteogenic differentiation of bone marrow MSC	
Skeletal muscle	*Bmal1*	*Myod1*, *Nr1d2*, *Rora*, *Dbp*, *Ppargc1b*, *Sox6*, *Mef2a*, *Six1*		Skeletal muscle function and phenotype maintaining; Skeletal muscle rhythm maintaining; Muscle-specific and fiber-type gene adjustment	[34, 84]
	*Clock*	*Myod1*, *Ppargc1b*		Skeletal muscle function and phenotype maintaining; Mitochondrial volume and metabolic function maintaining	[34]

*Rora*: RAR-related orphan receptor A; *Sox6*: SRY-box transcription factor 6; *Mef2a*: myocyte enhancer factor 2A; *Six1*: SIX homeobox 1. MSC, mesenchymal stem cell. *represent the possible genes associated with rhythm in the specific tissue

**Table 2:** Phenotypes of global and continual KO of rhythmic genes in periarticular tissues

Tissue	Gene type	Phenotype	Reference
Cartilage	*Bmal1*^−/−^	Osteoarthritic chondrocyte with high catabolism	[36, 44, 49]
	*Per2*^−/−^	Chondrocyte with high anabolism	[49]
Subchondral bone	*Bmal1*^−/−^	Bone resorption osteoblast	[29]
	*Bmal1*^−/−^	Osteoclast with low bone resorption ability	[31]
Synovium	*Bmal1*^−/−^	Inflammatory cell line	[32]
Skeletal muscle	*Bmal1*^−/−^	Premature aging muscle cell	[90]

KO, knockout

Cartilage, as a stress-bearing and spreading structure, is a time-sensitive tissue. The thickness of cartilage increases from night to morning and decreases from morning to night [17]. Alternations to cartilage thickness have been identified to be more obvious in the morning than in the evening during exercise [18]. This is associated with the internal rhythmic genes of chondrocytes, including *Bmal1*, *Clock*, *Per2*, *Cry1*, *Nr1d1* and *Nr1d2*. These genes are mostly controlled by core rhythmic genes, which consist of positive and negative regulatory arms [19]. The genes in the positive arm mainly include *Bmal1* and *Clock*, and the genes in the negative arm mainly include *Per2* and *Cry1* [20] [21]. The expression of *Bmal1* and *Clock* produces the BMAL1/CLOCK dimer. The BMAL1/CLOCK dimer is an activator of *Per2* and *Cry1*, which in turn depress the expression of *Bmal1* and *Clock*. When the expression of *Bmal1* and *Clock* decreases, the level of the BMAL-1/CLOCK dimer declines. Then, the expression of *Per2* and *Cry1* is down-regulated, which dismisses the depression of *Bmal1* and *Clock*. Therefore, these core rhythmic genes maintain a relatively fixed frequency of oscillation and facilitate the establishment of the rhythmic fluctuations of other rhythmic genes, such as *Cd44*, matrix metallopeptidase 13 (*Mmp13*), tissue inhibitor of metalloproteinase 1 (*Timp1*) and insulin-like growth factor 1 (*Igf1*). These genes are involved in cartilage matrix synthesis and cartilage degradation [22]. Additionally, the normal oscillatory expression of rhythmic genes is essential for keeping the balance between the anabolic and catabolic metabolism of chondrocytes. For instance, *Bmal1*, as a key rhythmic gene, depresses the depressor of the transforming growth factor beta (*Tgfb*) pathway, elastin (*Eln)* and tenascin (*Tnc*), and it enhances the expression of *Tgfb* to defend against chondrocyte hypertrophy through the SMAD family member 3 (*Smad3*) pathway, which induces chondrogenesis through mesenchymal condensation, as well as the proliferation of chondroblasts and the deposition of cartilage-specific ECM molecules [23]. Cryptochrome 2 (*Cry2*) maintains a strict rhythmic fluctuation in the chondrocytes via the inhibition of *Nr1d1*, *Nr1d2*, D-box binding protein (*Dbp*) and TEF transcription factor, as well as the PAR bZIP family member (*Tef*), to maintain the cartilage matrix and cartilage rhythm [24].

In subchondral bone, direct evidence of the relationships between normal rhythmic oscillations and subchondral bone homeostasis is insufficient [25]. Osteoclasts, osteoblasts and mesenchymal stem cells (MSCs) are comprised of subchondral bone and have been reported to be associated to the biological rhythms of joint tissues [25]. Multiple rhythmic genes maintain an oscillatory expression in bone marrow MSCs, including *Bmal1*, *Clock*, *Cry1*, period 1 (*Per1*), *Per2*, period 3 (*Per3*), glycogen synthase kinase 3b (*Gsk3b*), *Nr1d1*, *Nr1d2* and *Dbp* [26, 27]. *Clock* promotes the adipocytes differentiation of bone marrow MSCs by activating fatty acid binding protein 4 (*Fabp4*) expression (28). At the same time, *Gsk3b* promotes the adipogenesis of MSCs via the upregulation of adipocytic maturation associated genes, *Fabp4*, peroxisome proliferator activated receptor gamma (*Pparg*) and CCAAT enhancer binding protein alpha (*C/ebpalpha*). *Gsk3b* also enhances the osteogenic differentiation of MSCs by inducing alkaline phosphatase (*Alp*) expression, and it adjusts the cell cycle of bone marrow MSCs by maintaining the content of proteins in cell cycle regulation, including P19, P27, CYCLIN B1 and CYCLIN D1 [28]. *Per2* is an essential rhythmic gene for maintaining the oscillatory expression of *Gsk3b*, and it is also involved in the osteogenic differentiation and adipogenesis of bone marrow MSCs by maintaining the normal expression of *C/ebpalpha* and *Osteocalcin* [28]. Additionally, the expression of *Bmal1* and *Per1* result in a rhythmic oscillation in osteoblasts. The normal expression of *Bmal1* maintains bone mineral density and volume by depressing bone resorption marker genes, including matrix metallopeptidase 9 (*Mmp9*), cathepsin K (*CatK*), triiodothyronine receptor auxiliary protein (*Trap*), TNF receptor superfamily member 11a (*Rank*), receptor activator of nuclear factor kB ligand *(Rankl*) and calcitonin receptor (*Calcr*) [29]. *Bmal1* also inhibits the osteoclastogenesis of osteoblasts by repressing 1a,25-dihydroxyvitamin D3-induced *Rankl* to balance the resorption rate in bone [29]. Rhythmic gene *Per1* regulates the deposition of small apatite crystals of osteoblasts to maintain the rhythmic oscillation of mineralization in bone tissue [30]. In osteoclasts, rhythmic gene *Bmal1* maintains the number of osteoclasts in bone tissue by maintaining the normal expression of the osteoclastic gene *Nfatc1* [31].

Biological rhythm is also important in the homeostasis of synovium and skeletal muscle. For instance, the normal expression of *Bmal1* arrests the inflammation of synovium by the expression of anti-inflammatory factors interleukin 10 (*Il10*), interferon gamma (*Ifng*) and interleukin 13 (*Il13*), which reduce the production of inflammatory cytokines interleukin 6 (IL6), C-X-C motif chemokine ligand 1 (CXCL1), C–C motif chemokine ligand 2 (CCL2) and C-X-C motif chemokine ligand 5 (CXCL5) in fibroblast-like synoviocytes (FLS) [32]. Additionally, rhythmic gene *Clock* is involved in the prevention of fibroblasts, as well as macrophages in synovium, from OA inflammation and the accumulation of tumor necrosis factor (TNFA) [33]. In normal conditions, skeletal muscle has its own relatively constricted rhythms. Multiple genes are involved in such skeletal muscle rhythms and the homeostasis of skeletal muscle. Core rhythmic genes, *Clock* and *Bmal1*, are essential for maintaining skeletal muscle function and its phenotype. These two core rhythmic genes collaborate to establish the rhythmic oscillation and normal expression of myogenesis differentiation 1 (*Myod1*) at the transcription level [34]. In addition, the normal expression of *Clock* and *Bmal1* upregulates fibronectin type III domain containing 5 (*Fndc5*), vascular endothelial growth factor A (*Vegfa*), annexin A5 (*Anxa5*), thrombospondin 1 (*Thbs1*) and insulin-like growth factor binding protein 4 (*Igfbp4*) in skeletal muscle to maintain its metabolic homeostasis and normal rhythm. Also, *Clock* and *Bmal1* downregulate growth differentiation factor 11 (*Gdf11*) to preserve the function and construction of skeletal muscle [35].

## Rhythm disturbance in OA cartilage

**Fig. 1:**
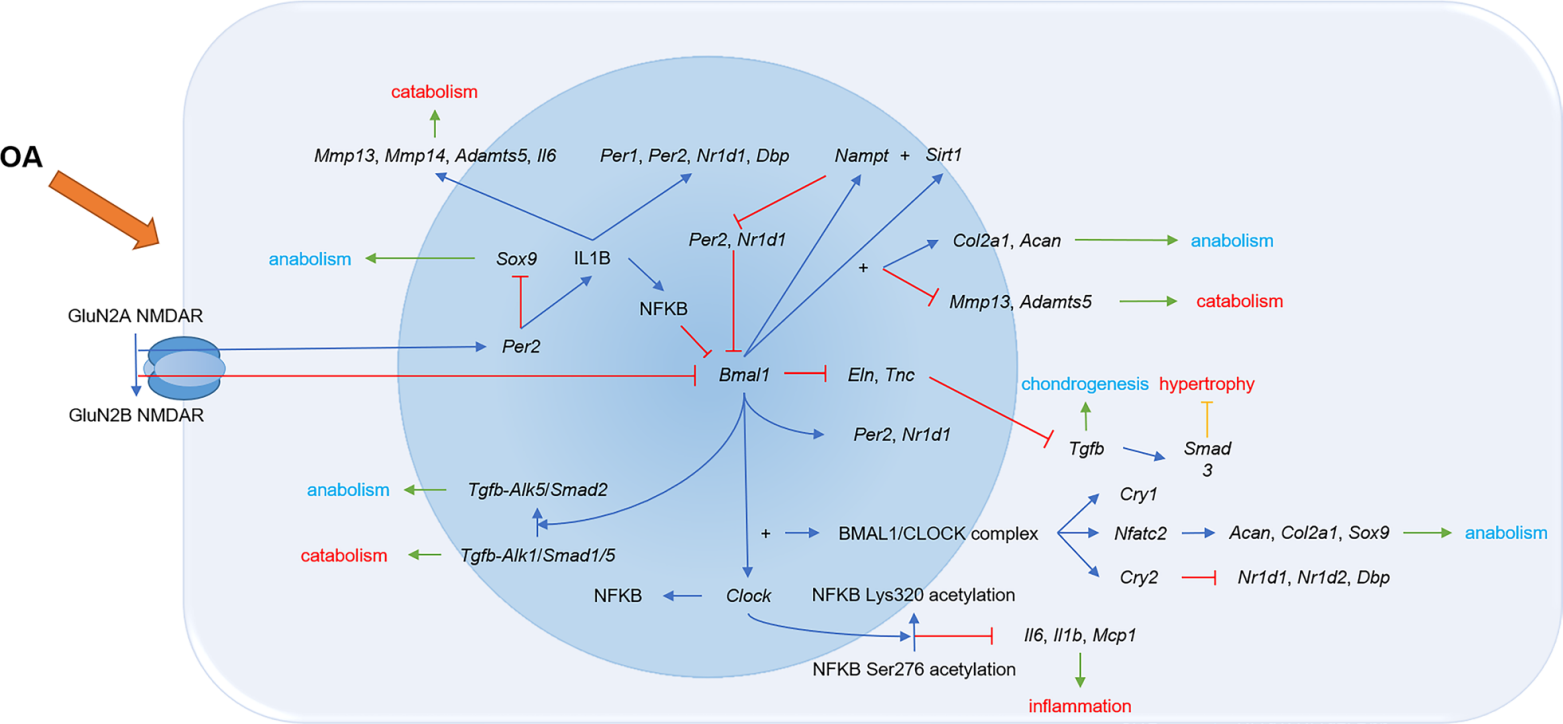
The rhythmic disturbance and its consequences in chondrocytes in osteoarthritis condition. During osteoarthritis process, the cartilage core rhythmic genes, including *Bmal1*, *Clock* and *Per2* are disturbed. The disturbance of core rhythmic genes in cartilage increases the catabolism and decreases the anabolism through Tgfb pathway. Other rhythm-related genes, including *Col2a1*, *Mmp13*, *Adamts5* are also associated to metabolic disorder in osteoarthritis cartilage. OA, osteoarthritis; *Bmal1*: brain and muscle arnt-like protein 1; *Clock*: circadian locomotor output cycles kaput; *Per1*: period 1; *Per2*: period 2; *Cry1*:cryptochrome 1; *Mmp13*: matrix metallopeptidase 13; *Mmp14*: matrix metallopeptidase 14; *Adamts5*: ADAM metallopeptidase with thrombospondin type 1 motif 5; *Il6*, interleukin 6; *Nr1d1*, nuclear receptor subfamily 1 group D member 1; *Nr1d2*, nuclear receptor subfamily 1 group D member 2; *Dbp*, D-box binding protein; *Nampt*, nicotinamide phosphoribosyltransferase; *Sirt1*, sirtuin 1; *Sox9*, SRY-box transcription factor 9; IL1B, interleukin 1 beta; NFKB: nuclear factor kappa B; *Col2a1*, type II collagen alpha 1 chain; *Acan*, aggrecan; *Eln*, elastin; *Tnc*, tenascin; *Tgfb*, transforming growth factor beta; *Smad3*, SMAD family member 3; *Nfatc2*, nuclear factor of activated T cells 2; *Alk5/Smad2*, transforming growth factor beta receptor 1/SMAD family member factor 2; *Alk1/Smad1/5*, ALK receptor tyrosine kinase/SMAD family member factor 1/5; *Mcp1*, monocyte chemoattractant protein 1

**Fig. 2:**
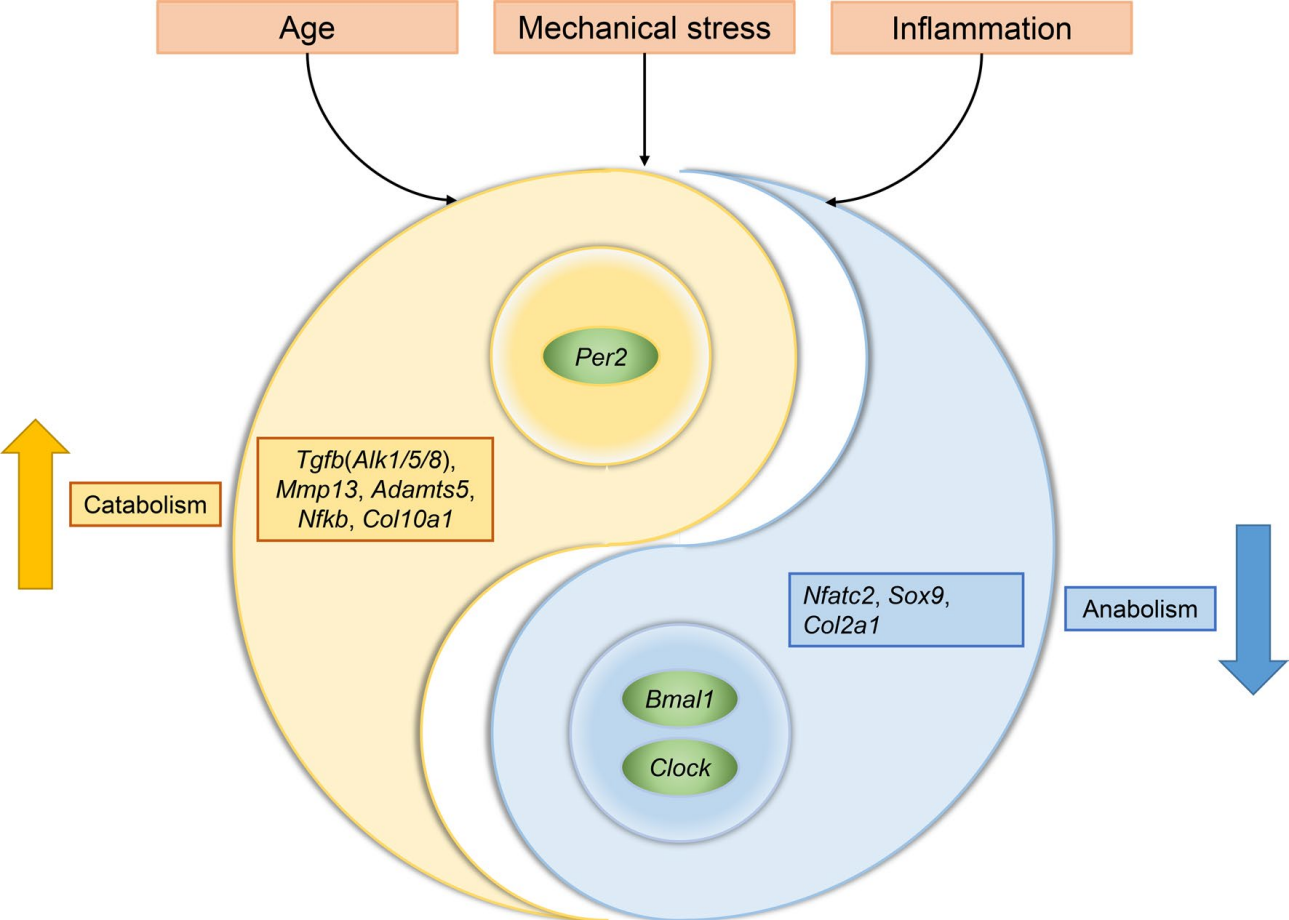
Metabolic disorder in osteoarthritis cartilage associated with rhythmic disturbance. Age, abnormal mechanical stress, and inflammation in osteoarthritis progression up regulate the catabolism by enhancing the core rhythmic gene *Per2*, and down regulate the anabolism by depressing the core rhythmic genes *Bmal1* and *Clock*. *Bmal1*: brain and muscle arnt-like protein 1; *Clock*: circadian locomotor output cycles kaput; *Per2*: period 2; *Nfatc2*, nuclear factor of activated T cells 2; *Sox9*, SRY-box transcription factor 9; *Col2a1*, type II collagen alpha 1 chain; *Mmp13*: matrix metallopeptidase 13; *Adamts5*: ADAM metallopeptidase with thrombospondin type 1 motif 5; *Nfkb*: nuclear factor kappa B; *Tgfb*, transforming growth factor beta; *Col10a1*, collagen type X alpha 1 chain

OA is a degenerative joint disease that is thought to stem from biomechanical stressors and biochemical changes [2, 3]. Cartilage degeneration and damage are its main pathological manifestation and initiate the start-up of inflammation in the tissues surrounding joints. Interestingly, it has been reported that this pathological progression interferes with cartilage rhythms. For instance, chondrocytes in OA change the expression of glutamate ionotropic receptor NMDA type subunit 2A (*Grin2a*) to glutamate ionotropic receptor NMDA type subunit 2B (*Grin2b*). This causes the reduced expression of *Bmal1* and SRY-box transcription factor 9 (*Sox9*), as well as the overexpression of *Per2*, collagen type X alpha 1 chain (*Col10a1*) and *Mmp13*, which thus further aggravates cartilage damage and rhythmic disorder [36]. When this biological rhythm is disturbed, catabolic enzyme genes become overexpressed, including *Mmp13* and ADAM metallopeptidase with thrombospondin type 1 motif 5 (*Adamts5*) involved in protein kinase C (PKC) and the extracellular signal regulated kinase (ERK) mitogen activated protein kinase (MAPK) axis, RUNX family transcription factor 2 (*Runx2*) and nuclear factor kappa B (*Nfkb*) pathways. These procatabolic substances RUNX2 and NFKB enhance cartilage degeneration in reverse. On the other hand, chondrogenesis genes like *Sox9* and tissue inhibitor of metalloproteinase 3 (*Timp3*) are depressed in OA cartilage as a result of circadian rhythm disruptions (Fig. 1) [19, 37–39]. In a constant 24-h darkness experiment, cartilage matrix synthesis genes lost rhythmicity, including aggrecan (*Acan*), type II collagen alpha 1 chain (*Col2a1*) and lysyl oxidase (*Lox*). Also, cartilage degrading genes membrane type 1 matrix metalloproteinase (Mt1-mmp/Mmp-14) and meningioma expressed antigen 5 (*Mgea5*) were up-regulated in 24-h darkness compared with 12 h of light and 12 h of darkness [40]. Moreover, cartilage rhythm disorders were aggravated by immune, inflammatory, hormone and other factors as well (Fig. 2) [37].

With OA progression, the expression of rhythmic genes in the positive arm, including *Bmal1* and *Clock*, is depressed. For instance, the level of *Bmal1* and the number of chondrocytes with normal *Bmal1* expression decrease sharply in OA cartilage [41]. This is related to an abundance of inflammatory factor interleukin 1 beta (IL1B) in damaged cartilage. IL1B has been reported to interfere with the expression of core rhythmic genes *Bmal1* through the NFKB signaling pathway [42]. The depression of *Bmal1* interferes with cartilage metabolism and affects cartilage rhythms negatively. While *Bmal1* is depressed in OA chondrocytes, the inhibition of *Eln* and *Tnc* is relieved. The overexpression of *Eln* and *Tnc* down-regulates the *Tgfb* pathway, which plays an essential role in chondrogenesis [23]. On the other hand, the *Tgfb* pathway in chondrocytes switches from transforming growth factor beta receptor 1/SMAD family member factor 2 (*Alk5/Smad2*), known as the chondrocytes’ anabolic pathway, to ALK receptor tyrosine kinase/SMAD family member factor 1/5 (*Alk1/Smad1/5*), known as the chondrocytes’ catabolic pathway, and it also exacerbates cartilage degeneration [41, 43–45]. When *Bmal1* and sirtuin 1 (*Sirt1*) are depressed in OA, the expression of cartilage anabolic genes *Col2a1* and *Acan* decreases sharply, and the catabolic genes *Mmp13* and *Adamts5* increase, which eventually causes the degeneration of cartilage [46]. Meanwhile, the depression of *Bmal1* reduces the volume of the putative E-box–containing region of the Nfatc2 loci as well as the expression of the nuclear factor of activated T cells 2 (*Nfatc2*) due to a reduction of the CLOCK/BMAL1 complex [41, 42]. *Nfatc2* is the key chondrocyte transcription factor for maintaining the healthy homeostasis of chondrocytes [41]. Along with this reduction of *Nfatc2* mRNA, inflammatory and catabolic pathways are activated through *Mmp13* signaling, and anabolic signaling factors like *Acan*, *Col2a1* and *Sox9* are depressed, which aggravates the degeneration of cartilage [47]. The reduction of CLOCK/BMAL1 also depresses the expression of *Clock*, *Per1*, *Per2*, *Nr1d1*, *Dbp*, *Cry1* and *Cry2*, resulting in a disorder of the chondrocytes’ rhythms [41, 42]. Interestingly, with the decrease of *Bmal1* in OA, the activity of nicotinamide phosphoribosyltransferase (*Nampt*) and the expression of *Sirt1* are inhibited, which increases the level of *Per2* and *Nr1d1*, thus causing a decrease of *Bmal1* in turn [46]. Along with the disorder of *Bmal1* expression of chondrocytes in OA, *Clock* expression is also disrupted. With excessive mechanical stress in OA joint, *Clock* is depressed, which inhibits *Nfkb* at the transcriptional level [20, 48]. Moreover, with the mutation of *Clock*, the acetylation of NFKB at the Lys310 residue is inhibited, and the phosphorylation of NFKB at the Ser276 residue is promoted, which leads to the over activation of NFKB and inflammatory factors such as IL6, IL1B and monocyte chemoattractant protein 1 (MCP1); this also activates the chondrocyte inflammatory program [48]. As the rhythmic genes of the positive arm, *Clock* and *Bmal1* are both depressed in the chondrocytes of OA, and *Per2*, the rhythmic gene in the negative arm, is upregulated. The overexpression of *Per2* leads to a decrease of the anabolic agent SOX9 level and an increase of catabolic agents MMP13 and ADAMTS5 through the *Il1b* pathway [49].

Moreover, the intrinsic rhythmic genes in chondrocytes are partly controlled by the central rhythmic system through hormones. For example, melatonin, as a circadian information translator secreted by the pineal body, reduces cartilage degradation [50]. Melatonin affects cartilage rhythm by upregulating *Per2* and *Cry1* expression (51). In vitro, a low dose of melatonin increases the expression of *Per2* and maintains the chondrocyte proliferation in a TNFA cultured environment [51]. Melatonin also restrains the pathological catabolic shifting of the cartilage in OA through the down-regulation of catabolic genes, such as vascular endothelial growth factor (*Vegf*), *Mmp13* and *Alp* [51]. Also, melatonin protects chondrocytes from oxidative stress-induced cytotoxicity and inflammatory mediators via the assistance of Sirt1 by inhibiting the expression of nitric oxide synthase (*Inos*) and cytochrome c oxidase subunit 2 (*Cox2*), as well as their production, in addition to nitric oxide (NO), prostaglandin E2 (PGE2), TNFA, IL1B and interleukin 8 (IL8) [52].

## Rhythm disturbance in OA subchondral bone

**Fig. 3:**
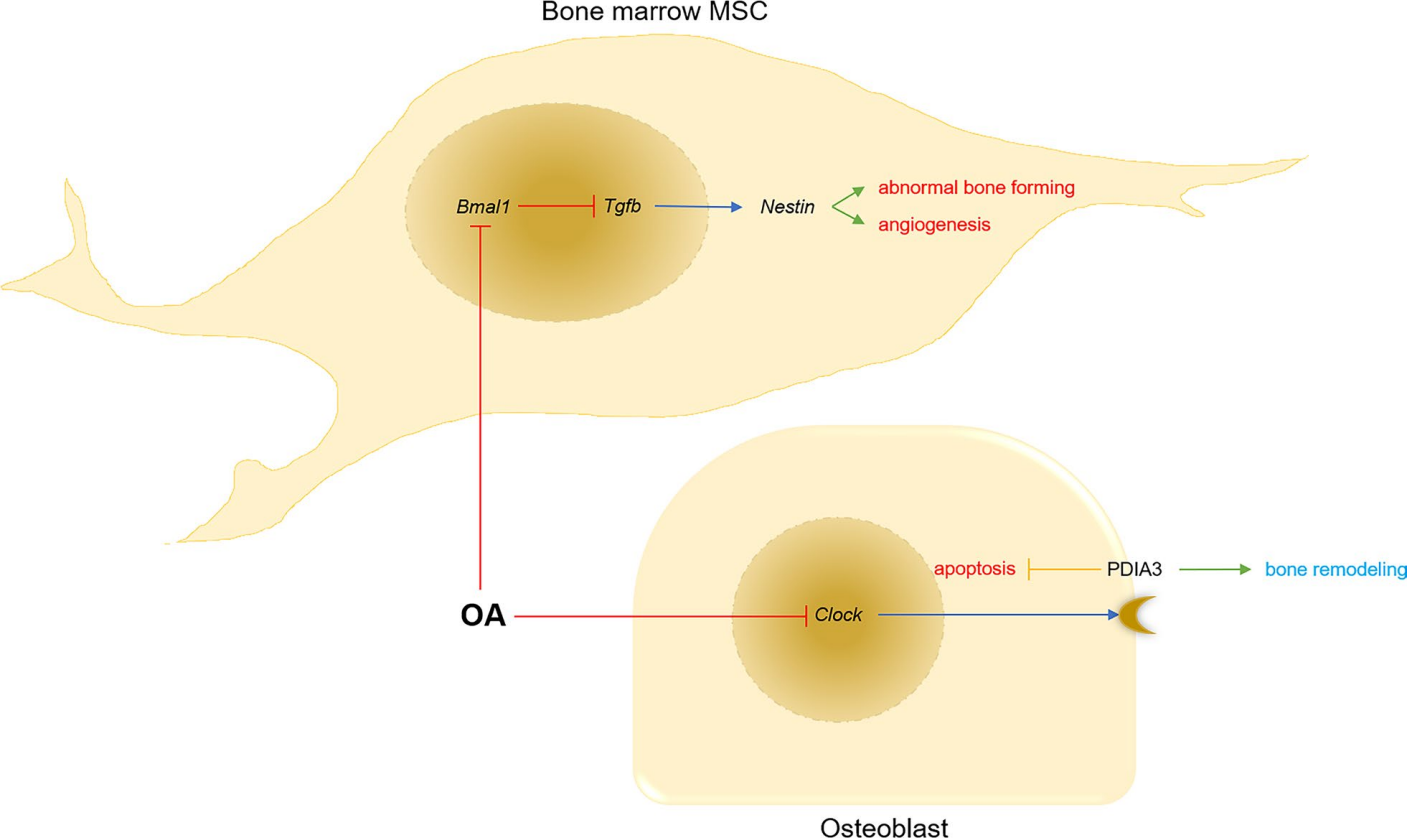
The rhythm disturbance and its consequences in subchondral bone in osteoarthritis condition. During osteoarthritis process, the core rhythmic gene, *Bmal1*, is disturbed in bone marrow mesenchymal stem cells, which aggravates the abnormal bone forming with high level of angiogenesis in subchondral bone. Meanwhile, the core rhythmic gene, *Clock*, is disturbed in subchondral bone osteoblasts, which promotes the apoptosis of osteoblasts and inhibits bone remodeling. OA, osteoarthritis; MSC, mesenchymal stem cell; *Bmal1*: brain and muscle arnt-like protein 1; *Tgfb*, transforming growth factor beta; *Clock*: circadian locomotor output cycles kaput; PDIA3, protein disulfide isomerase family A member 3

Subchondral bone is also involved in OA pathological change [53, 54]. In OA joint, the expression of rhythmic genes is disrupted, which affects bone remodeling due to a decrease in bone formation and an increase in cell apoptosis, thus leading to the thinning of subchondral bone (Fig. 3) [55]. Also, aberrant rhythms in subchondral bone lead to a reduction of cartilage repairability and an acceleration of cartilage damage [19, 56].

Disorders of rhythmic genes, including *Bmal1*, *Clock* and *Cyr2*, are related to subchondral bone dysfunction in OA joint. For instance, a deficiency of the core rhythmic gene *Bmal1* activates the *Tgfb* pathway in subchondral bone tissue and induces the formation of nestin + MSC clusters, finally causing aberrant bone formation accompanied by high levels of angiogenesis [22]. The activation of the *Tgfb* pathway also promotes OA progression through abnormal osteoblasts [23, 57]. In addition, the decreased expression of *Bmal1* disturbs the ossification of para-articular tissue, leading to the calcification and ossification of the periarticular tendon and ligaments related to bone insertion sites [58]. Also, the reduction of *Bmal1* negatively affects the growth of the longitudinal bone. Low expression levels of *Bmal1* in epiphysis block the hypoxia inducible factor 1 subunit alpha (*Hif1a*) pathway, resulting in a decrease of its downstream production, VEGF [59]. This causes decreased vascular ingrowth in epiphysis, which is essential for the calcification and ossification process of endochondral bone growth [60]. Except for the disruption of *Bmal1* expression, *Clock* is also reduced in OA joints. The down-regulation of *Clock* decreases the transcription level of the protein disulfide isomerase family A member 3(PDIA3) as a 1α,25(OH)2D3 receptor. When PDIA3 is relatively deficient, the compensatory effect of *Clock* expression in osteoblasts is reduced, resulting in the apoptosis of osteoblasts and bone remodeling abnormalities; as a consequence, bone density drops [61, 62]. *Cry2* is another important rhythmic gene for maintaining subchondral bone homeostasis. When *Cry2* is depressed in OA joints, subchondral bone gains an increased number of blood vessels, and more severe damage is incurred [24].

Meanwhile, the rhythm and homeostasis of subchondral bone is controlled by the CNS through circadian-secreted hormones (Table 3). Melatonin, known as an important rhythmic agent secreted by the pineal body, regulates the rhythms in bone tissue to inhibit the function of osteoclasts and to maintain normal bone metabolism [63]. Also, melatonin promotes bone-marrow-derived MSCs chondrogenesis, especially in the early stages of differentiation. Melatonin up-regulates chondrogenic genes, including *Acan*, *Col2a1* and *Col10a1*, in MSCs during chondrogenic differentiation. Transcription factors SOX9 and RUNX2, which are essential to chondrogenesis, are also potentiated in melatonin-treated MSCs [64]. Melatonin also enhances the cartilage differentiation of bone marrow-derived MSCs through elevated miR-590-5p and miR-526b-3p, along with SMAD1 phosphorylation, by targeting SMAD family member 7 (*Smad7*) [65]. Meanwhile, melatonin subdues the apoptosis of the MSCs in bone marrow, and it upregulates chondrogenic markers, including *Col2a1*, *Acan*, *Sox9* and *Col10a1*, upon the presence of IL1B [66]. The secretion of the parathyroid hormone (PTH) follows a relatively strict rhythmic oscillation. PTH can reset the rhythmic oscillation of *Per2* in the growth plate of the femur through parathyroid hormone 1 receptor (PTH1R), thus maintaining the normal rhythms of epiphysis [67]. Thyroid stimulating hormone (TSH) is another rhythm-related hormone associated with bone health. TSH enhances osteoblast function by phosphorylating AKT serine/threonine kinase 1 (AKT1) and ERK1/2, as well as by upregulating osteoblast marker genes, *Alp*, *Rankl* and *Osteocalcin* [68]. TSH also attenuates *Tnfa* expression and inhibits the c-Jun NH2-terminal kinase/jun protooncogene (*Jnk*/*c-jun*) and *Nfkb* signal pathways to thus decrease osteoclasts genesis and thereby downregulate bone remodeling and reduce bone loss [68–72]. In addition, cortisol, as a diurnally secreted steroid hormone, is capable of increasing bone fracture risk and worsening OA pathology through the potentiation of the expression of enzyme hydroxysteroid 11-beta dehydrogenase 1 (*Hsd11b1*) in osteoblasts and osteocytes [73, 74].

**Table 3:** Possible hormones and their function involved in homeostasis of subchondral bone

Hormone	Secretory organ	Target cell	Involved pathway	function	Reference
Melatonin	Pineal body	Bone marrow MSC	*Acan*, *Col2a1*, *Col10a1*, *Sox9*, *Runx2*	Chondrogenesis	[64]
TSH	Adenohypophysis	Osteoblast	*Alp*, *Rankl*, *Osteocalcin*	Osteoblastogenesis	[69]
		Osteoclast	*Jnk/c-jun*, *Nfkb*	Inhibition of osteoclastogenesis	[71]
Cortisol	Adrenal gland	Osteoblast	*Hsd11b1*	Decrease in bone formation	(74)

TSH, thyroid stimulating hormone

## Rhythm disturbance in synovium and skeletal muscle

**Fig. 4:**
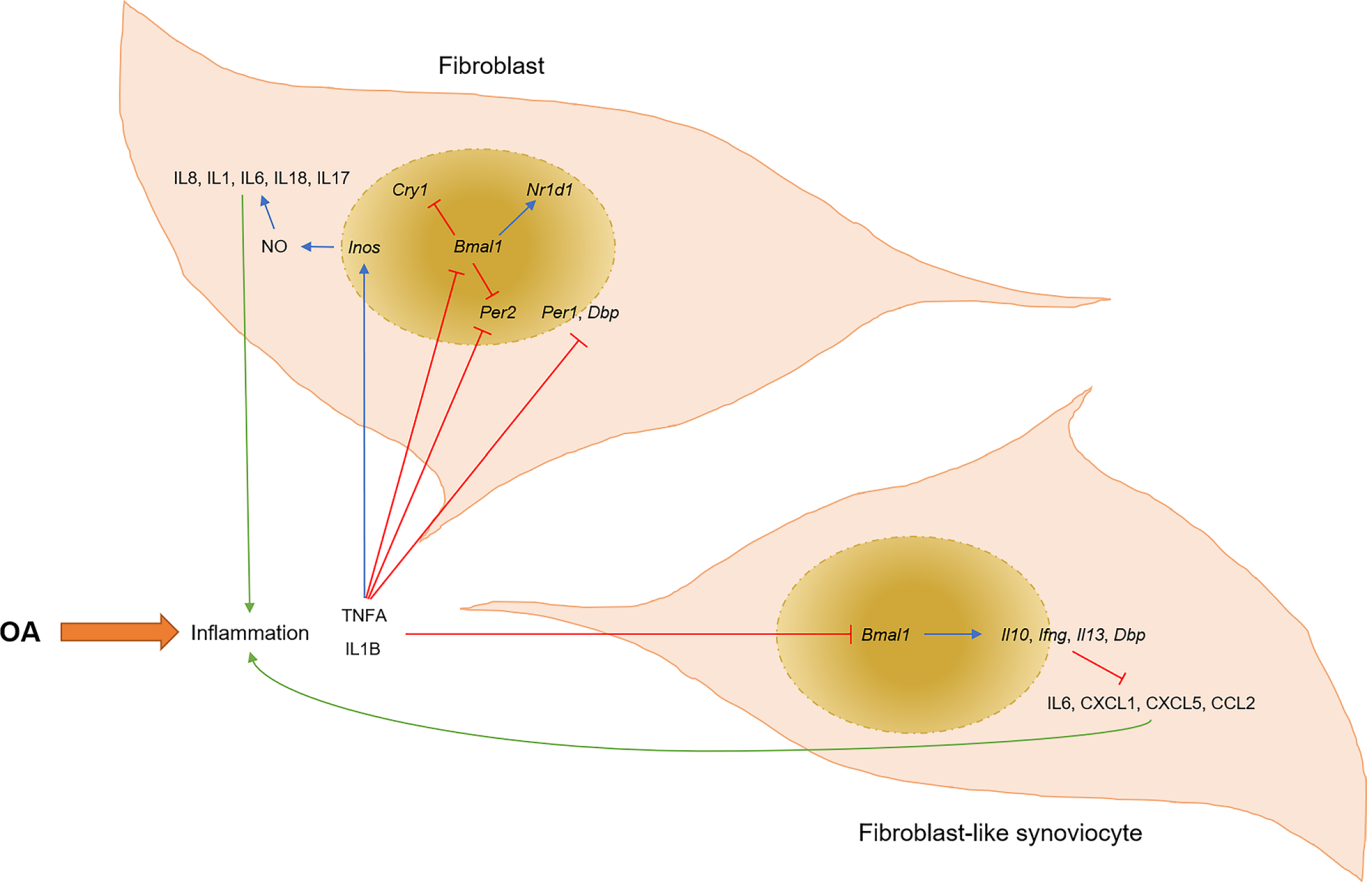
The rhythm disturbance and its consequences in synovium in osteoarthritis condition. During osteoarthritis process, the core rhythmic gene, *Bmal1*, is disturbed in fibroblasts and fibroblast-like synoviocytes, which aggravates the arrhythmicity and inflammation in synovium. OA, osteoarthritis; TNFA, tumor necrosis factor; IL1B, interleukin 1 beta; *Inos*: nitric oxide synthase; NO, nitric oxide; IL8, interleukin 8; IL1, interleukin 8; IL6, interleukin 6; IL18, interleukin 18; IL17, interleukin 17; *Bmal1*: brain and muscle arnt-like protein 1; *Cry1*, cryptochrome 1; *Per1*, period 1; *Per2*, period 2; *Dbp*, D-box binding protein; *Nr1d1*, nuclear receptor subfamily 1 group D member 1; *Il10*, interleukin 10; *Ifng*, interferon gamma; *Il13*, interleukin 13; CXCL1, C-X-C motif chemokine ligand 1; CXCL5, C-X-C motif chemokine ligand 5; CCL2, C–C motif chemokine ligand 2

The synovium is a periarticular tissue with good blood perfusion. Immune cells can migrate into the joint through the synovial membrane and produce cytokines associated with joint inflammation in the joint space and the circulatory system [75]. Concomitantly, inflammatory cytokines in the circulatory system can permeate through the synovium into the space of the joint [76]. The synovium follows a normal rhythm in a healthy joint, but in OA conditions, inflammation disturbs the rhythm of the synovium (Fig. 4). During the OA process, the synovium secretes great amounts of inflammatory factors, including IL1B and TNFA. IL1B and TNFA are able to promote the production of NO through the *Inos* pathway and induce the formation of pro-inflammatory factors, including IL8, interleukin 1 (IL1), IL6, interleukin 18 (IL18) and interleukin 17 (IL17) [77]. Also, when TNF is enriched, the transcription of intrinsic rhythmic genes *Dbp*, *Per1* and *Per2* is depressed in the synovial fibroblasts of the OA synovium, causing a loss of rhythm in synovial fibroblasts [78, 79]. At the same time, enriched TNFA and IL1B depress the expression of *Clock* in the OA synovium, and the inflammation of the joint is exacerbated by synovitis [33, 37]. While inflammatory cytokines disrupt the rhythms of the synovium and aggravate the severity of OA, the disorganization of its daily rhythm promotes the infiltration of mast cells in the synovium, thus inducing a low-grade inflammatory condition, which is a hallmark of OA [39]. Additionally, rhythmic disorders activate catabolic mediators, phospho-PKC, phospho-ERK1/2 and MMP13 in the synovium, resulting in a OA pathological change to the joint [39]. Moreover, the disruption of rhythmic genes such as *Bmal1*, *Per2* and *Cry1* is responsible for arrhythmicity and inflammation in the synovium of an OA joint. When *Bmal1* is depressed in an OA synovium, the diurnal oscillations of *Dbp* and *Nr1d1* are lost, and non-oscillatory expressions of *Per2* and *Cry1* are enhanced, leading to synovial rhythm disorder [32]. Simultaneously, the depression of *Bmal1* causes the thickening of the synovium subintima due to synovium fibrosis [32]. Also, the low expression of *Bmal1* in synovial FLS renders the resident immune cells, including neutrophils and Ly6ChiHi-monocytes, more sensitive in response to challenge, which promotes mononuclear cell infiltration and raises the cytokine production in the OA joint [80]. Other rhythmic genes, such as *Cry2*, are damped in an OA joint; as a consequence, the inflammation in the synovium is much more severe [24].

The rhythm and homeostasis of the synovium are controlled by the central rhythm of the CNS through hormones. For instance, melatonin follows a relatively rhythmic oscillation during the day and plays a role in preserving the homeostasis of the synovium [81]. Melatonin inhibits the inflammatory factor *Il1b* pathway and reduces the intracellular reactive oxygen species (ROS) in synovial MSCs. With the reduction of ROS, the proliferation capacity and viability of synovial MSCs are improved [82]. At the same time, melatonin promotes the bone differentiation process and the production of ALP, type I collagen and osteocalcin in synovial MSCs [82].

**Fig. 5:**
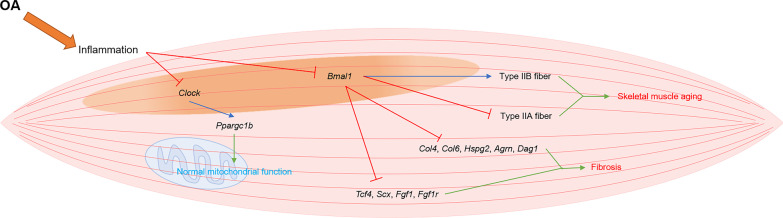
The rhythm disturbance and its consequences in skeletal muscle cell in osteoarthritis condition. During osteoarthritis process, the skeletal muscle cell core rhythmic genes, including *Bmal1* and *Clock* are disturbed. The disturbance of core rhythmic genes in skeletal muscle cell aggravates the aging, fibrosis, and mitochondria dysfunction. OA, osteoarthritis; *Bmal1*: brain and muscle arnt-like protein 1; *Clock*: circadian locomotor output cycles kaput; *Col4*, collagens 4; *Col6*, collagens 6; *Hspg2*, proteoglycan perlecan; *Agrn*, agrin; *Dag1*, dystroglycan; *Tcf4*, fibroblast associated genes transcription factor 4; *Scx*, scleraxis; *Fgf1*, fibroblast growth factor 1; *Fgf1r*, Fgf1 receptor; *Pparg*, peroxisome proliferator activated receptor gamma

Skeletal muscle is a periarticular structure that maintains the stability of joints. Skeletal muscle shares multiple common rhythmic genes with cartilage, including *Per2*, *Bmal1* and *Cry1* [19]. During the OA process, periarticular skeletal muscle is a vulnerable tissue due to OA inflammation, and its rhythms are attenuated (Fig. 5). In an OA joint, the rhythmic oscillations and homeostasis of skeletal muscle are destroyed due to the high levels of IL6 released by the infrapatellar fat pad. This leads to weakness in the periarticular skeletal muscle and in turn promotes OA progression [77, 83].

In OA joints, the rhythmic genes of skeletal muscle, including *Bmal1* and *Clock*, are disrupted, resulting in the aging and weakness of skeletal muscle. The reduction of *Bmal1* is related to the aging of skeletal muscle. The volume of type IIB fibers in skeletal muscle decreases along with an increased volume of the more oxidative type IIA fibers in *Bmal1*–knocked out skeletal muscle [84]. In addition, the loss of *Bmal1* promotes fibrosis in skeletal muscle due to the overexpression of extracellular matrix genes collagens 4 (*Col4*), collagens 6 (*Col6*), proteoglycan perlecan (*Hspg2*), agrin (*Agrn*), dystroglycan (*Dag1*), fibroblast associated genes transcription factor 4 (*Tcf4*), scleraxis (*Scx*), fibroblast growth factor 1 (*Fgf1*) and the Fgf1 receptor (*Fgf1r*) [84]. Moreover, this weakness of the skeletal muscle is associated with the down-regulation of *Bmal1*. The down-regulation of *Bmal1* causes an attenuation of the diameter and amount of skeletal muscle fiber [85]. On the other hand, low expression levels of *Bmal1* interfere with the highly conserved hexagonal arrangement of thin and thick filaments in skeletal muscle, which then obtain lower specific tension [34]. Aberrant *Bmal1* expression also affects the volume and function of mitochondria in skeletal muscle, especially beneath the skeletal muscle membrane. With an approximately 40% reduction in *Bmal1* mutant skeletal muscle cells, the remaining mitochondria present a pathological status characterized by swelling and the disruption of the cristae. In addition to their irregular morphology, the respiratory control ratio of mitochondria in *Bmal1* knocked-out skeletal muscle is also damped due to a reduction in state 3 respiration [34]. The number and functional alternation of mitochondria is also associated to the blocking of PPARG coactivator 1 beta (*Ppargc1b*) through the reduction of *Clock* [34]. Separately, the expression of *Nr1d1* also retains a rhythmic oscillation in skeletal muscle. When *Nr1d1* is disturbed in skeletal muscle, myogenic differentiation and muscle regeneration are inhibited, and the autophagy of skeletal muscle cells is activated [86, 87].

Moreover, the rhythms and homeostasis of skeletal muscle are controlled by the central rhythms of the CNS via hormones. For example, melatonin rescues the rhythmic disruption of skeletal muscle in the OA condition by up-regulating the expression of *Bmal1* and *Clock* [51]. Melatonin also rebuilds the normal expression of myosin heavy chain 4 (*Myh4*), a myosin heavy chain IIB protein encoding gene, in skeletal muscle in OA condition [51].

## Conclusions

The joint is a time-sensitive organ that is partly controlled by the central rhythm in the CNS and that has its own peripheral rhythm [88]. The normal expression of rhythmic genes protects the cartilage, synovium, subchondral bone and skeletal muscle of the joint from the pathological alternation of OA. In addition, the regular central rhythm of the CNS guarantees the ordinary oscillation of rhythmic gene expression in joint tissues through hormones. With the burden of OA and disturbances to the CNS rhythm, however, intrinsic rhythmic genes such as *Bmal1*, *Clock*, *Per1*, *Per2* and *Nr1d* are disturbed in multiple periarticular tissues, which in turn aggravates the progression of OA [89].

In cartilage, disorders of rhythmic gene expression increase the catabolism and decrease the anabolism of chondrocytes. Along with the aberrant metabolism in chondrocytes, cartilage degenerates, and the OA process accelerates. In subchondral bone, the mutation of rhythmic genes aggravates the dysfunction of osteoblasts and osteoclasts, leading to an abnormal remodeling of subchondral bone tissue. Moreover, this rhythmic disorder interferes with the chondrogenesis of bone-marrow derived MSCs and retards cartilage repairment. In the synovium, abnormal rhythmic gene expression inhibits the chondrogenesis of synovial MSCs and potentiates inflammation, resulting in progressive cartilage damage that worsens the pain and dysfunction of the joint. In skeletal muscle, rhythmic disturbances accelerate the aging of skeletal muscle fibers and interfere with the volume and function of mitochondria in skeletal muscle cells, which induces skeletal muscle weakness. In this case, the stability of the joint decreases, and the pathological change of OA accelerates.

**Fig. 6:**
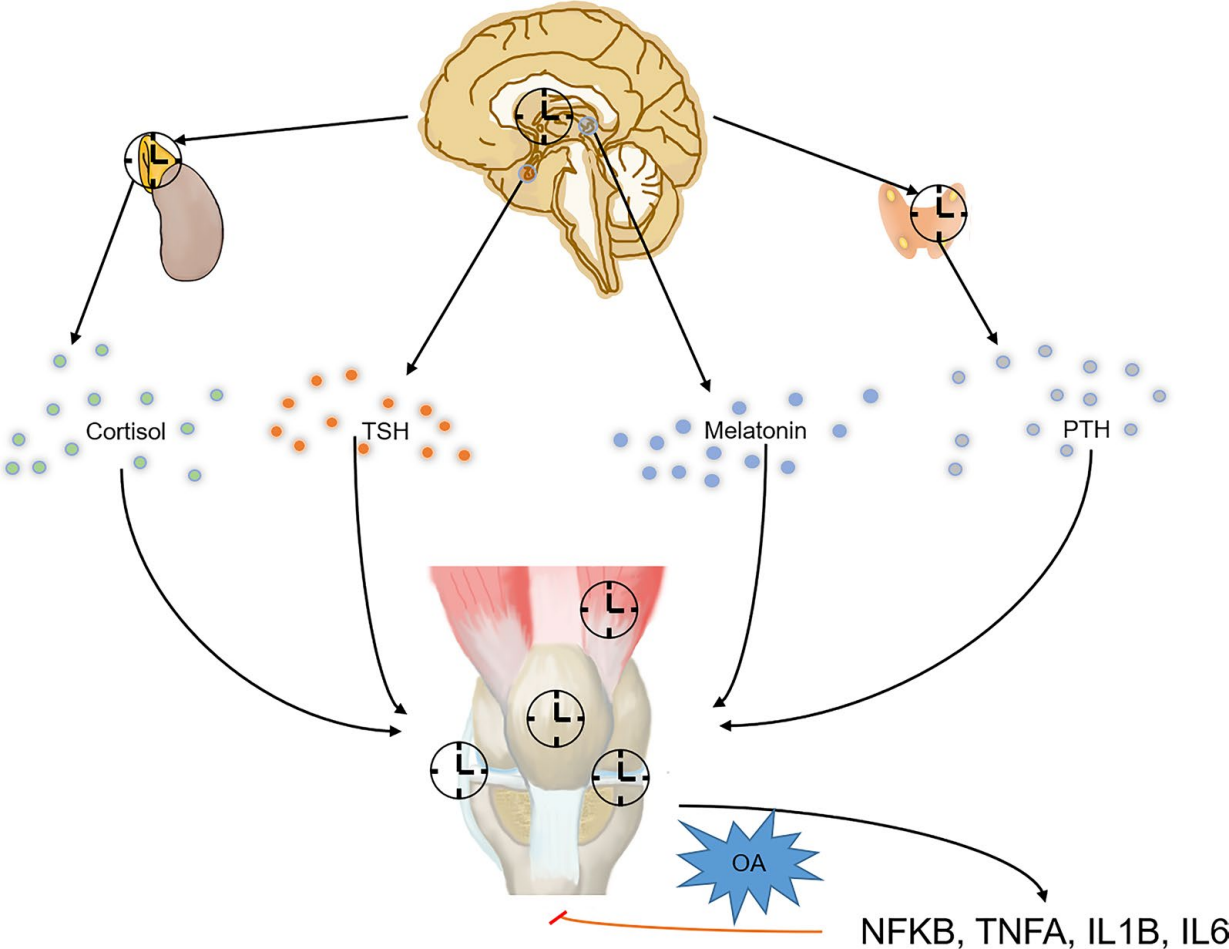
Crosstalk between the joint rhythm and the central nervous system rhythm. Central nervous system is able to adjust the joint rhythm through hormones, including cortisol, thyroid simulating hormone, melatonin, and parathyroid hormone. With osteoarthritis progression, the central rhythm is interrupted by the symptom of osteoarthritis joint. This in turn affects the normal rhythm in periarticular tissues, and aggravates osteoarthritis pathological change. TSH, thyroid simulating hormone; PTH, parathyroid hormone; OA, osteoarthritis; NFKB, nuclear factor kappa B; TNFA, tumor necrosis factor; IL1B, interleukin 1 beta; IL6, interleukin 6

Rhythm-related genes are not the only factors responsible for the aggravation of cartilage damage, synovitis and dysfunction in OA joints. The rhythmic disturbance of hormone secretion due to CNS rhythmic disorders also plays an important role in the rhythmic gene dysfunction of periarticular tissues; this, in turn, activates immune cells and raises inflammation cytokines in the articular space. Rhythmic regulators, such as melatonin, corticoid and TSH, modify intrinsically rhythmic genes in periarticular tissues in case of inflammation and damage. These rhythmic mediators also have a circadian secretion phase under normal conditions. With rhythmic disturbances caused, however, by shift work and irregular sleep/activity schedules, the rhythmic secretion of these hormones is affected, which can lead to the development of cartilage degeneration, synovitis and osteoporosis (Fig. 6). Also, the abnormal nerve insertion of the OA joint through the subchondral bone and synovia may play a role in the circadian disruption and diurnal symptoms of the joint.

Research focused on rhythmic disturbances and OA provide new conceptions of pathological changes in OA joints and make it possible to study new drugs for treating OA via these mechanisms. The challenge will be to further characterize key rhythmic genes, their regulators and the downstream pathways involved in OA pathological change, as well as to manufacture medicine targeting these genes.

## Data Availability

Not applicable.

## Abbreviations

OA: Osteoarthritis
CNS: Central nervous system;
*Bmal1*: Arnt-like protein 1
*Clock*: Circadian locomotor output cycles kaput
*Per1*: Period 1
*Per2*: Period 2
*Cry1*: Cryptochrome 1
SCN: Suprachiasmatic nucleus
COMP: Cartilage oligomeric matrix protein
HA: Hyaluronan
KS5D4: Keratan sulfate
TGFB1: Transforming growth factor b1;
CTXII: Urinal C-telopeptide of Collagen II
PIIANP: Procollagen type IIA N-terminal propeptide
HELIXII: Type II collagen helical peptide
Nr1d1: Nuclear receptor subfamily 1 group D member 1
*Nr1d2*: Nuclear receptor subfamily 1 group D member 2
*Mmp13*: Matrix metallopeptidase 13
*Timp1*: Tissue inhibitor of metalloproteinase 1
*Igf1*: Insulin-like growth factor 1
*Tgfb*: Transforming growth factor beta
*Eln*: Elastin
*Tnc*: Tenascin
*Smad3*: SMAD family member 3
*Cry2*: Cryptochrome 2
*Dbp*: D-box binding protein
*Tef*: TEF transcription factor, as well as the PAR bZIP family member
MSCs: Mesenchymal stem cells
*Per1*: Period 1
*Per3*: Period 3
*Gsk3b*: Glycogen synthase kinase 3b
*Fabp4*: Fatty acid binding protein 4
*Pparg*: Peroxisome proliferator activated receptor gamma
*C/ebpalpha*: CCAAT enhancer binding protein alpha
*Alp*: Alkaline phosphatase
*Mmp9*: Matrix metallopeptidase 9
*CatK*: Cathepsin K
*Trap*: Triiodothyronine receptor auxiliary protein
*Rank*: TNF receptor superfamily member 11a
*Rankl*: Receptor activator of nuclear factor kB ligand
*Calcr*: Calcitonin receptor
Il10: Interleukin 10 (Il10)
*Ifng*: Interferon gamma
*Il13*: Interleukin 13
*Il6*: Interleukin 6
CXCL1: C-X-C motif chemokine ligand 1
CXCL5: C–C motif chemokine ligand 2 (CCL2) and C-X-C motif chemokine ligand 5
FLS: Fibroblast-like synoviocytes
TNFA: Tumor necrosis factor
*Myod1*: Myogenesis differentiation 1
*Fndc5*: Fibronectin type III domain containing 5
*Vegfa*: Vascular endothelial growth factor A
*Anxa5*: Annexin A5
*Thbs1*: Thrombospondin 1
*Igfbp4*: Insulin-like growth factor binding protein 4
*Gdf11*: Growth differentiation factor 11
*Grin2a*: Glutamate ionotropic receptor NMDA type subunit 2A
*Grin2b*: Glutamate ionotropic receptor NMDA type subunit 2B
*Sox9*: SRY-box transcription factor 9
*Col10a1*: Collagen type X alpha 1 chain
*Adamts5*: ADAM metallopeptidase with thrombospondin type 1 motif 5
PKC: Protein kinase C
ERK: Extracellular signal regulated kinase
MAPK: Mitogen activated protein kinase
*Runx2*: RUNX family transcription factor 2
*Nfkb*: Nuclear factor kappa B
*Timp3*: Tissue inhibitor of metalloproteinase 3
*Acan*: Aggrecan
*Col2a1*: Type II collagen alpha 1 chain
*Lox*: Lysyl oxidase
*Mt1-mmp/Mmp-14*: Membrane type 1 matrix metalloproteinase
*Mgea5*: Meningioma expressed antigen 5
IL1B: Interleukin 1 beta
*Alk5/Smad2*: Transforming growth factor beta receptor 1/SMAD family member factor 2
*Alk1/Smad1/5*: ALK receptor tyrosine kinase/SMAD family member factor 1/5
*Sirt1*: Sirtuin 1
*Nfatc2*: Nuclear factor of activated T cells 2
*Nampt*: Nicotinamide phosphoribosyltransferase
MCP1: Monocyte chemoattractant protein 1
*Vegf*: Vascular endothelial growth factor
*Inos*: Nitric oxide synthase
*Cox2*: Cytochrome c oxidase subunit 2
NO: Nitric oxide
PGE2: Prostaglandin E2
IL8: Interleukin 8
*Hif1a*: Hypoxia inducible factor 1 subunit alpha
PDIA3: Protein disulfide isomerase family A member 3
*Smad7*: SMAD family member 7
PTH: Parathyroid hormone
PTH1R: Parathyroid hormone 1 receptor
TSH: Thyroid stimulating hormone
AKT1: AKT serine/threonine kinase 1
*Jnk/c-jun*: C-Jun NH2-terminal kinase/jun protooncogene
*Hsd11b1*: Hydroxysteroid 11-beta dehydrogenase
IL1: Interleukin 1 (IL1)
IL18: Interleukin 18
IL17: Interleukin 17
*Col4*: Collagens 4
*Col6*: Collagens 6
*Hspg2*: Proteoglycan perlecan
*Agrn*: Agrin
*Dag1*: Dystroglycan
*Tcf4*: Fibroblast associated genes transcription factor 4
*Scx*: Scleraxis
*Fgf1*: Fibroblast growth factor 1
*Fgf1r*: Fgf1 receptor
*Ppargc1b*: PPARG coactivator 1 beta
*Myh4*: Myosin heavy chain 4
KO: Knockout
